# Fine-structural distribution of MMP-2 and MMP-9 activities in the rat skeletal muscle upon training: a study by high-resolution in situ zymography

**DOI:** 10.1007/s00418-012-0940-5

**Published:** 2012-03-15

**Authors:** Marine Yeghiazaryan, Katarzyna Żybura-Broda, Anna Cabaj, Jakub Włodarczyk, Urszula Sławińska, Marcin Rylski, Grzegorz M. Wilczyński

**Affiliations:** 1 Nencki Institute of Experimental Biology, Polish Academy of Sciences, Pasteura 3, 02-093 Warsaw, Poland; 2 Institute of Biocybernetics and Biomedical Engineering, Trojdena 4, 02-109 Warsaw, Poland; 3 The Medical Center of Postgraduate Education, Marymoncka 99/103, 01-813 Warsaw, Poland

**Keywords:** Matrix metalloproteinase, Exercise, Skeletal muscle, Rat, Cell nucleus, Satellite cells

## Abstract

Matrix metalloproteinases (MMPs) are key regulators of extracellular matrix remodeling, but have also important intracellular targets. The purpose of this study was to examine the activity and subcellular localization of the gelatinases MMP-2 and MMP-9 in skeletal muscle of control and physically trained rats. In control hind limb muscle, the activity of the gelatinases was barely detectable. In contrast, after 5 days of intense exercise, in Soleus (Sol), but not Extensor digitorum longus (EDL) muscle, significant upregulation of gelatinolytic activity in myofibers was observed mainly in the nuclei, as assessed by high resolution in situ zymography. The nuclei of quiescent satellite cells did not contain the activity. Within the myonuclei, the gelatinolytic activity colocalized with an activated RNA Polymerase II. Also in Sol, but not in EDL, there were few foci of mononuclear cells with strongly positive cytoplasm, associated with apparent necrotic myofibers. These cells were identified as activated satellite cells/myoblasts. No extracellular gelatinase activity was observed. Gel zymography combined with subcellular fractionation revealed training-related upregulation of active MMP-2 in the nuclear fraction, and increase of active MMP-9 in the cytoplasmic fraction of Sol. Using RT-PCR, selective increase in MMP-9 mRNA was observed. We conclude that training activates nuclear MMP-2, and increases expression and activity of cytoplasmic MMP-9 in Sol, but not in EDL. Our results suggest that the gelatinases are involved in muscle adaptation to training, and that MMP-2 may play a novel role in myonuclear functions.

**Electronic supplementary material:**

The online version of this article (doi:10.1007/s00418-012-0940-5) contains supplementary material, which is available to authorized users.

## Introduction

It is generally accepted that skeletal muscle is a dynamic structure, which can be profoundly remodeled by different patterns of motoneuronal activity. Skeletal muscle plasticity is characterized by the ability to modify structure, and alter mechanical and metabolic properties, in response to exercise, electrical stimulation, diseases or microgravity (Bassel-Duby and Olson 2006; Fluck 2006; Ohira 2000).

Alteration of physical activity induces significant changes in gene expression pattern, which, in turn, regulates structure of the extracellular matrix and myofibers, cytoskeleton and membranes protein composition, as well as metabolic enzymes to improve efficiency of muscle function in a new type of activity (Fluck 2006; Goldspink 2003; Pilegaard et al. 2000). In the last decade, a vast amount of work has extended our understanding of the important role of extracellular matrix (ECM) molecules in exercise-induced muscle remodeling and shown the contribution of matrix metalloproteinases in muscle fiber adaptation to the training (Carmeli et al. 2004; Koskinen et al. 2004; Rullman et al. 2009).

Matrix metalloproteinases are key regulators of the formation, remodeling, and degradation of extracellular matrix in both physiological and pathological processes. The existence of MMPs has been reported in most tissues. In the skeletal muscle, MMPs were shown to be implicated in muscle physiology (development, angiogenesis, adaptive changes due to exercise and disuse) and various pathologies, including neuromuscular diseases, muscle dystrophy (Renaud and Leppert 2007; Zanotti et al. 2007), and ischemia (Baum et al. 2007). Because of their ability to degrade native type-IV, -V and -VII collagens, as well as denatured interstitial collagens (e.g. gelatin), MMP-2 and -9 (called gelatinases) are considered to play a major role in exercise-induced ECM remodeling (Collier et al. 1988; Visse and Nagase 2003; Wilhelm et al. 1989). It is, however, unknown, what are the cellular sources of gelatinases in the muscle tissue upon exercise, and what their subcellular distribution is. Interestingly, recent studies showed that these enzymes, in addition to being extracellularly secreted, can be active inside the cell, and may have substrates that are not extracellular matrix components (Cauwe and Opdenakker 2010; Kwan et al. 2004). For example, MMP-2 is found in distinctive subcellular compartments, including thin and thick myofilaments of the cardiac sarcomere (Sawicki et al. 2005; Wang et al. 2002), cytoskeleton (Coker et al. 1999; Sung et al. 2007), nuclei (Cauwe and Opdenakker 2010; Kwan et al. 2004), and mitochondria (Wang et al. 2002), and was shown to be able to cleave troponin I, myosin light chain-I and α-actinin during myocardial oxidative stress injury (Wang et al. 2002).

In the present study, we investigated the cellular and subcellular localization of MMP-2 and -9 in the rat slow-twitch fiber rich Sol and fast-twitch fiber rich EDL skeletal muscles upon physical exercise. Unexpectedly, we found mainly nuclear distribution and activation of MMP-2 in the trained Sol muscle, whereas MMP-9 was associated with the cytoplasmic compartment, especially in activated satellite cells/myoblasts.

## Methods

### Animals and training

A total of 54 male Wistar rat, aged between 2 and 3 months (mean weigh 200 ± 50 g) were used in this study. The animals had free access to water and chow and were kept in collective cages (4 rats per cage). The research was approved by the Local Ethics Committee in Warsaw. All animal procedures complied with European Union and Polish Law on Animal Protection.

The animals were randomly assigned to the following three groups: control-untrained (*n* = 11), experimental-acutely exercised (*n* = 20) and experimental-trained (*n* = 23), to be analyzed by different techniques (see below). Control-untrained animals were kept in their cages without any type of exercise. The animals were subjected to an acute exercise session or were trained for 5, 14 and 30 days.

After 3 days of familiarization period with the laboratory and motor-driven treadmill, the trained group animals started the running session. During first day of running, training time and speed of the treadmill was increased gradually from 10 min at 7 cm/sek to 45 min at 20 cm/sek at a 0 % grade. After this time the animals were able to run for 45 min a day at 20 cm/sek with no interruptions.

### Tissue processing

The muscles subjected to morphological studies (immunohistochemistry, and in situ zymography) were isolated from 4 control, 12 acutely exercised, and 12 trained rats. To obtain the muscles, the animals were anesthetized by intraperitoneal injection of sodium pentobarbital (60 mg/kg body weight) and perfused for 2–3 min via ascending the aorta with 0.5 % paraformaldehyde in 0.1 M phosphate-buffered saline (PBS), pH 7.2.

Muscle samples were dissected before the training (control group) and 2, 6 and 24 h after the first exercise session (acute exercised group), or 5, 14 and 30 days after treadmill running (training group). After perfusion, Sol and EDL muscles from each animal were carefully dissected out from both hind limbs and cleared of fat and connective tissues. Next muscles from both legs were cryoprotected in 30 % sucrose in 0.1 M PBS (up to 48 h at 4 °C) and frozen in cold isopentane (~−80 to −90 °C). Frozen muscles were cryostat-sectioned at −20 °C into 14 μm thick longitudinal sections, immobilized on polylysine-covered glass slides and kept at −20 °C until analysis. The weak fixation (0.5 % PFA) and subsequent cryoprotection was found to be essential for preserving optimal morphology in subsequent in situ zymography (see below). These procedures do not affect the gelatinase activity, as compared to the sections of the snap-frozen tissue (not shown).

### In situ zymography

The cryostat sections of Sol and EDL were air dried at the room temperature and pre-incubated 3 × 5 min in dye-quenched (DQ) buffer with 0.01 % Triton X-100 from Sigma (buffer—supplied by the manufacturer or made according to the recipe in DQ gelatin manual; 10× concentrated: 0.5 M Tris–HCl, 1.5 M NaCl, 50 mM CaCl_2_, pH 7.6). Sections were then overlaid with a flurogenic substrate DQ gelatin (Invitrogen/Molecular Probes, Eugene, OR, USA) diluted 1:100 in the DQ buffer and incubated for 2 h at 37 °C. Slides were then rinsed 3 × 10 min in PBS containing 0.01 % Triton X-100. Cleavage of the substrate by gelatinases results in increase of fluorescence intensity by unblocking of quenched fluorescence. After washing, sections were used for immunohistochemical staining.

Negative controls were obtained by processing some of the sections with the potent zinc chelator 1,10-phenanthroline (1.4 mM, Invitrogen) or by prefixation of the sections in 4 % paraformaldehyde solution.

### Immunofluorescence

Prior to the labeling, the sections were blocked with 5 % normal donkey serum in 0.01 % PBS-Triton X-100 (NDST) for 60 min at room temperature to reduce non-specific staining. To visualize selected epitopes, specific antibodies were used: polyclonal rabbit anti-MMP-9 (Torrey Pines Biolabs), diluted 1:100, polyclonal rabbit anti-MMP-2, 1:200 (Santa Cruz), monoclonal mouse anti-Pax-7, 1:500 (Developmental Studies Hybridoma Bank, University of Iowa), monoclonal mouse M-Cadherin, 1:100 (BD Biosciences), monoclonal mouse anti-MyoD, 1:100 (BD Biosciences), monoclonal mouse anti-CD45 biotinolated, 1:100 (Abcam), monoclonal mouse anti-desmin, 1:25 (Sigma), monoclonal mouse anti-β-Dystroglican, 1:10 (Novocastra), polyclonal rabbit anti-RNA PolII (phospho S2) (1:100, Abcam). All the primary antibodies were diluted in 5 % blocking serum in 0.01 % PBS-Triton X-100 (PBST). Sections were covered with antibody solution and incubated overnight at 4 °C. Then the specimens were washed 3 × 10 min in PBST prior to 2-h room-temperature incubation with the respective secondary antibody, coupled with a range of Alexa fluorophores (Alexa 488 or Alexa 555 or Alexa 647, all from Molecular Probes) diluted 1:200. After several washes and air drying, the sections with the Vectashield mounting medium (Vector Laboratories) were covered with a coverslip and examined under the microscope. Nuclei were counterstained with 4,6-diamidino-2-phenylindole (DAPI) or TO-PRO-3 (Molecular Probes) following the secondary antibody incubation.

### Microscopy and image processing

The fluorophore-labeled specimens were examined under the Leica TCS SP5 confocal system, or Leica DRB widefield fluorescence microscope equipped with a digital camera. The quantitative analysis of the DQ-gelatin signal intensity over the myonuclei or sarcolemma was performed in the images of the specimens triple-labeled for (1) β-dystroglycan, a marker of sarcolemma (Alexa 555), (2) gelatinolytic activity (fluorescein-conjugated DQ-gelatin), and (3) nuclei (DAPI), acquired through the confocal microscope (Suppl. Fig. 1). The measurements were performed blindly, according to a semi-authomatic algorithm utilizing basic routines of ImageJ software (freely available image processing program, http://rsb.info.nih.gov/ij). Briefly, every image (the three-channel 24-bit RGB), in which red channel represented β-dystroglycan immunoreactivity, green channel represented DQ gelatin signal, and blue channel represented the nuclear DAPI staining, obtained through the 40× objective (zoom 1) was split into the three individual 8-bit images, corresponding to the different channels. To measure the myonuclear activity specifically, the red and blue channels were merged, to identify and manually erase all nuclei situated outside the sarcolemma, using the appropriate black drawing tool. Then the edited red–blue image was again split, and the channel containing only myonuclei was inverted, and subtracted from the DQ-gelatin-containing channel (green). As a result, the image representing only the myonuclear activity (everything else black) was generated, and subjected to the “analyze particles” function of the ImageJ, that produced the mean myonuclear activity, together with the number of nuclei in the image. The two different fields of view were recorded for every animal, yielding up to 100 nuclei per individual. Finally, the mean myonuclear activity ± standard error was calculated for every experimental group (*n* = the number of animals/group) (see Suppl. Fig. 1 to inspect the workflow of the measurements). To measure the activity of the sarcolemma, first the membrane segments associated with the myonuclei were erased from the aforementioned red–blue image because, due to the limited resolution, the sarcolemmal activity could not be reliably separated from the underlying myonuclear activity. The edited image was again split, and the channel containing specifically the segments of sarcolemma not associated with the myonuclei was inverted, and subtracted from the DQ-gelatin-containing channel (green). As a result, the image representing only the sarcolemmal activity (everything else black) was generated, and subjected to the “measure” function of the ImageJ that produced the mean sarcolemmal activity per unit area of the section. Finally, the mean sarcolemmal activity ± standard error was calculated for every experimental group (*n* = the number of animals/group) (see Suppl. Fig. 1 to analyze the workflow of the measurements). The results were statistically analyzed using ANOVA.

Highest-resolution confocal images were restored by three-dimensional (3D) deconvolution using Huygens Professional software (Scientific Volume Imaging, Hilversum, Netherlands, http://www.svi.nl/) by applying classic maximum-likelihood estimation algorithm and automatically generated point-spread function. For final inspection, the images were processed using Corel Package (Corel Corporation, Ottawa, Ontario, Canada).

### Nuclear and cytoplasmic extraction

For subcellular fractionation, nuclear and cytoplasmic fractions of Sol muscle of control (*n* = 3), and 5-day trained rats (*n* = 3) were obtained using a commercially available kit (NE-PER Nuclear and Cytoplasmic Extraction Reagents—Thermo Fischer Scientific Inc., USA). Protein concentration was spectrophotometrically determined in the fractions using BCA protein assay reagent and bovine serum albumin as the standard. To assess the purity of the nuclear and cytoplasmic fractions, immunoblot analysis with anti-GAPDH, as cytoplasmic, and anti-RNA polII as a nuclear marker was done.

### Gel zymography

Samples for analysis (12 μl) were diluted twice with a buffer (pH 6.8) containing 0.125 M Tris–HCl, 4 % sodium dodecyl sulfate (SDS), 20 % glycerol and 0.004 % bromophenol blue. Protein molecular weight standards (Sigma) and samples were electrophoretically separated with Protean II system (Bio-Rad, Hercules, CA), in SDS-PAGE Tris–glycine 8.0 % polyacrylamide gel containing 2 mg/ml of gelatin (Merck) at constant current of 40 mA. The geles were washed twice in 2.5 % Triton X-100 for 15 min to remove SDS and incubated for 2 days in 50 mM Tris–HCl, pH 7.5, 10 mM CaCl_2_, 1 μM ZnCl_2_, 1 % Triton X-100, and 0.02 % sodium azide at 37 °C. The gels were then stained with 0.1 % Coomassie blue G-250 for 3 h in 40 % 2-propanol and destained with a solution containing 5 % acetic acid until clear bands of gelatinolysis appeared on a dark background. Wet zymograms were digitized using a scanner.

### RNA isolation and RT-PCR

Total RNA was isolated from Sol and EDL muscles of control (*n* = 4), acute exercised (immediately and after 2 h of single running session, *n* = 4 for each period) and trained rats (5 days and 2 weeks, *n* = 4 for each period) using Tri reagent (Sigma-Aldrich) according to the manufacture’s directions. Primers and PCR reaction conditions were the same as described previously (Szklarczyk et al. 2002). GAPDH mRNA expression was used as a loading control.

### Statistical analysis

For each variable of interest, a one-way analysis of variance (ANOVA) was performed to compare the two groups: exercise training and control. In the event of a significant *F* ratio, a post hoc analysis was conducted to identify significant pair-wise differences. Post hoc tests were performed using Fisher’s exact test. Student *t* test was used for statistical evaluation of the mRNA data. Results were expressed as mean ± standard deviation (SD). In all analyses, statistical significance was established at *p* ≤ 0.05. Statistical analysis was performed using SPSS for Windows.

## Results

### Repeated training upregulates the gelatinolytic activity of the myofibers

To determine the localization of gelatinolytic activity in skeletal muscle, we used the technique of in situ zymography (ISZ). In addition, this technique was combined with immunolabeling. We analyzed rat Sol and EDL muscles that contain, respectively, mainly slow-twich and fast-twich fibers, and display different physiological and biochemical responses to physical activity (Buonanno and Rosenthal 1996; Irintchev and Wernig 1987).

**Fig. 1:**
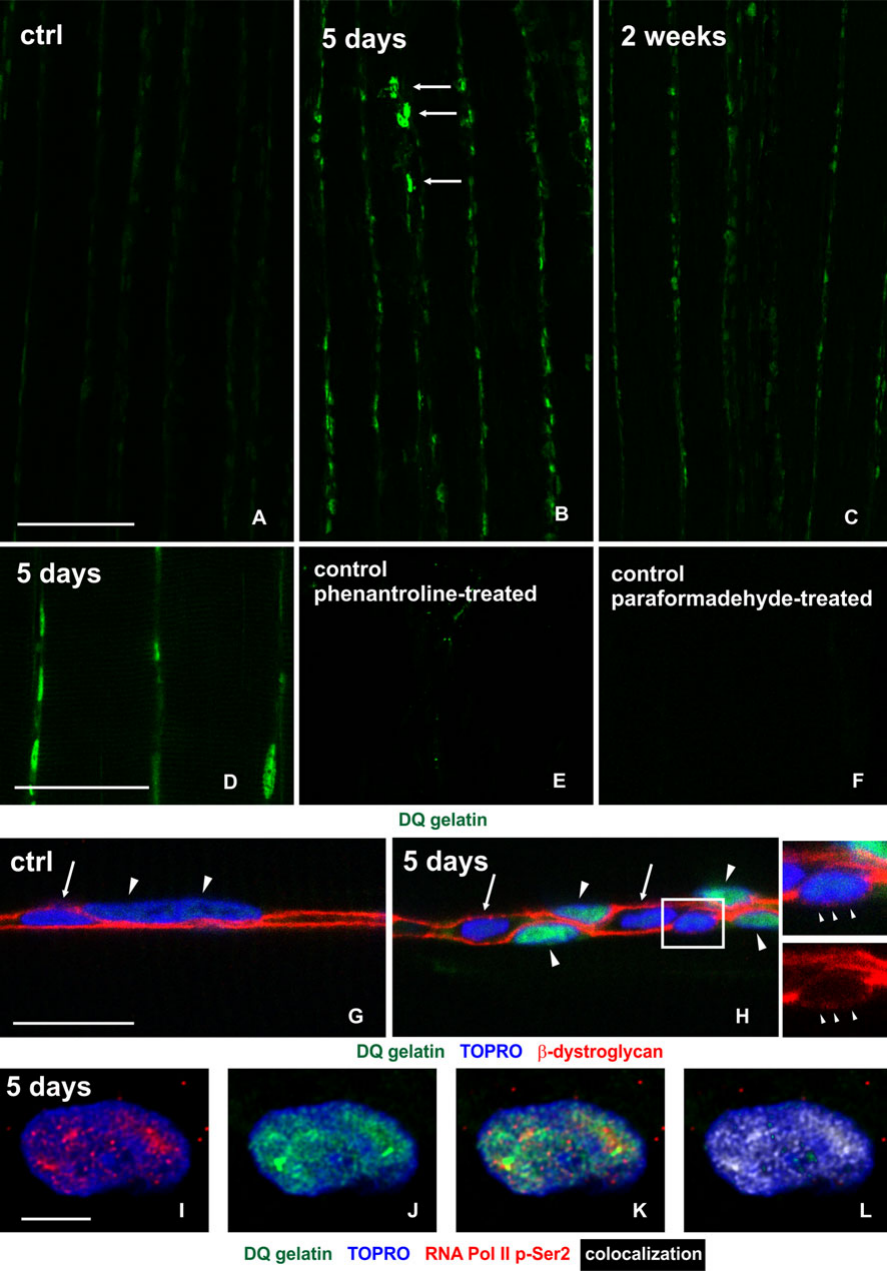
The pattern of gelatinolytic activity in control and trained rat Sol muscle. Low magnification confocal micrographs of in situ zymography in control muscle (**a**), after 5 days- (**b**), and after 2 weeks (**c**) of treadmill running. Note the exercise-induced prominent increase of the gelatinase activity (DQ-gelatin, *green*) in the cell nuclei. *Scale bar* 100 μm; Confocal images of in situ zymography performed in muscle of the animal trained for 5 days demonstrating the specificity of the assay using a zinc chelator 1,10-phenantroline (**e**), or prefixation of the section with 4 % PFA solution (**f**). Note the abolishment of gelatinase activity (*green*) in (**e**) and (**f**), compared to control zymography (**d**). *Scale bar* 30 μm. **g**, **h** Triple fluorescent staining for gelatinase activity (*green*), β-dystroglycan, a marker of sarcolemma (*red*), and DNA (TOPRO dye, *blue*) demonstrating that the gelatinolytic activity is present exclusively in myonuclei (*arrowheads*); the nuclei of endomysial connective tissue (*arrows*) do not have the gelatinolytic activity. *Inset* shows enlarged view of the gelatinase-negative nucleus outlined in (**h**), which appears to be attached to the muscle fiber, yet it is separated from it by a thin line of β-dystroglycan IR (*small arrowheads*); such a localization suggests that this is the nucleus of quiescent satellite cell. *Scale bar* 15 μm. High magnification confocal images of a myonucleus triple labeled for gelatinase activity (**j**, **k**, *green*), activated (e.g. transcribing) RNA polymerase II (phospho-Ser2 epitope) (**i**, **k**, *red*), and DNA (**i**–**l**, *blue*); Note the similarities in the distribution between *red* and *green signal* (compare **i** vs. **j**), and their extensive colocalization revealed as *yellow*/*orange* *color* in (**k**), or *white color* in (**l**); **l** represents an enhanced view of the colocalization: every pixel in which *red* and *green signals* overlap is labeled with *white*. The colocalization indicates that the gelatinase activity is spatially associated with the active regions of gene transcription. *Scale bar* 5 μm

When applied to untrained Sol and EDL muscle sections, ISZ revealed only weak gelatinolytic activity present throughout muscle fibers, with the inconspicuous labeling of the sarcoplasm and myonuclei indentified by DNA-specific staining (Fig. 1a–c, g, h). There were no apparent differences in signal intensity between fiber types, and the activity levels were similar in both muscles studied.

**Fig. 2:**
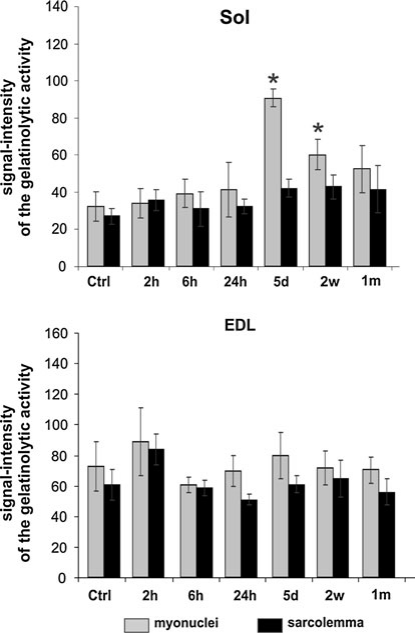
Quantitative evaluation of gelatinase activity in the muscle fibers of Sol (*upper panel*) and EDL (*lower panel*), at various time points throughout the experiment. The signal intensities were measured in myonuclei (*grey bars*) and sarcolemma (*black bars*) of control (Ctrl) and trained rats (2, 6, and 24 h after single bout of exercise, and after 5 days, 2 weeks and 1 month of repeated treadmill running). Data for each column were obtained from four male rats. Error bars represent standard error; *asterisk* indicates statistical significance, compared to control, *p* < 0.05 by ANOVA

The single bout of 45-min long exercise did not change the level of gelatinolytic activity in either Sol or EDL muscle, as measured at 2, 6 and 24 h after the run (Fig. 2). In contrast, 5 days of repeated physical training resulted in significant upregulation of gelatinolytic activity in myofibers of Sol, but not in EDL muscle (one-way ANOVA: *p* < 0.05) (Fig. 2). Notably, the labeling was increased mainly in the muscle nuclei, and to a lesser extent in the sarcolemma. The detailed investigation of nuclei with increased gelatinolytic activity, using β-dystroglycan immunoreactivity (IR) as a sarcolemma marker, confirmed that these were myonuclei, and not the nuclei of connective tissue cells (Fig. 1g, h).

Within the myonuclei, the gelatinolytic activity was distributed throughout the nuclear interchromatin area, and colocalized with the activated RNA Polymerase II phospoepitope (phosphoserine-2 of the C-terminal heptapeptide repeats) (Fig. 1i–l).

There was no gelatinolytic activity within the extracellular space. However, there were few foci of cells with strongly positive cytoplasm, located among myofibers, at the sites of apparent myofibers’ necrosis (Fig. 1b). These cells were identified as activated satellite cells/myoblasts (see below). The upregulation of gelatinase activity, with the very similar pattern, persisted in Sol muscle after 2 weeks of training (one-way ANOVA: p < 0.05), but returned to the control level after 1 month. The quantitative evaluation of fluorescent signals in major compartments of the muscle fibers of both Sol and EDL, at various time points throughout the experiment, is presented in Fig. 2.

The specificity of the staining was controlled by the use of MMP inhibitors such as: zinc chelator 1,10-phenanthroline, which is the general metalloproteinase inhibitor, or by prefixation of the sections in 4 % paraformaldehyde solution, which abolishes or greatly diminishes the fluorescent signal (Fig. 1d–f).

### MMP-2 and MMP-9 contribute differently to the upregulation of the gelatinolytic activity by exercise

**Fig. 3:**
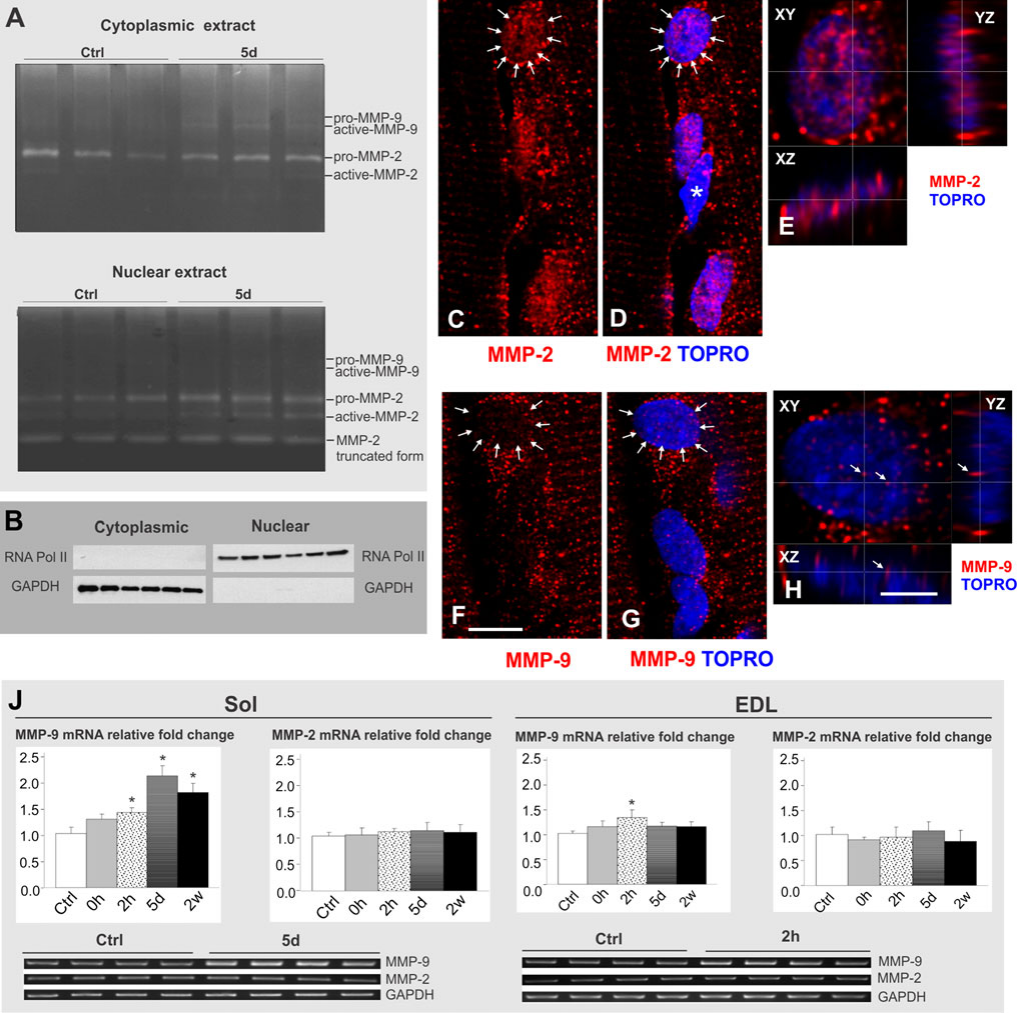
Activity, expression and localization of MMP-2 and MMP-9 upon training. **a** Gel zymography in cytoplasmic (*upper gel*) and nuclear (*lower gel*) fractions of Sol muscle from control and 5-day trained rats. In cytoplasmic fractions, there is a band corresponding to pro-MMP-2 that has an equal intensity in control and trained animals; treadmill running results in appearance of additional two bands representing pro- and active-MMP-9. Gel zymography of nuclear fraction in control reveals the presence of a prominent band corresponding to pro-MMP-2; training results in the enhancement of pro-MMP-2, and the appearance of a band representing active-MMP-2. In addition, in the nuclear fractions there is a band of lower molecular mass that most probably represents an autocatalytically derived active fragment of MMP-2. Each *lane* represents different animal. **b** Verification of purity and loading control of nuclear and cytoplasmic extracts from **a** by immunoblot analysis of GAPDH (cytoplasmic housekeeping protein) and RNA PolII (nuclear protein, non-phospho epitope). **c**–**h** Immunofluorescence analysis of subcellular distribution of MMP-2 (**c**; and *red channel* in **d**, **e**) and MMP-9 (**f**, and *red channel* in **d**, **e**). It is evident that MMP-2, but not MMP-9 immunoreactivity is present in the myonuclei identified by DNA-specific dye (TOPRO, *blue*). The images in (**c**, **d**, **f**, **g**) are maximum projections of a few focal planes. *Arrows* in (**c**, **d**, **f**, **g**) point to the myonuclei that are shown enlarged and cross sectioned in (**e**, **h**). *Asterisk* indicates the nucleus that does not belong to the muscle fiber and is MMP-2-negative; such nuclei do not contain the gelatinase activity either (see Fig. 1). In **e**, note that MMP-2 immunoreactivity fills the whole nucleus, as shown by single-plane cross sections of the entire confocal stack in all three dimensions (*XY*, *XZ*, *YZ*). In **h**, note that the tiny MMP-9-positive puncta (*arrows*) visible over the nuclear area in *XY* view do not belong to the nucleus, they are cytoplasmic structures closely adjacent to it, as demonstrated by *XZ* and *YZ* cross sections. The apparent presence of these structures in the *XY* image is due to the fact that the microscope has lower resolution in *Z* dimension, compared to *X* and *Y* dimensions. *Scale bars* for (**c**, **d**, **f**, **g**) and (**e**, **h**) are, respectively, 10 and 5 μm. **j** RT-PCR analysis of MMP-2 and MMP-9 mRNAs in Sol and EDL muscles. Training results in a prominent increase of MMP-9 mRNA in Sol muscle at 2 h after single bout of exercise that remains after 5 days and 2 weeks of treadmill running (*left panel*). MMP-9 mRNA is increased also in EDL muscle at 2 h after single bout of exercise (*right panel*), *p < 0.05, Student’s *t* test (*n* = 4)

Using in situ zymography technique alone, it is not possible to distinguish between MMP-2 and MMP-9 activities because the DQ-gelatin is cleaved by both enzymes. To find out the identity of gelatinase activity within muscle cell cytoplasm and nuclei after physical training, we used gel zymography (an electrophoretic technique) combined with subcellular fractionation (Fig. 3a). Since the previous quantitative evaluation of ISZ results showed the peak of activity in Sol muscle after 5 days of treadmill running, the gel zymography was performed in nuclear and cytoplasmic fraction of Sol at that time point. To assess the relative purity of the fractions and to control for loading, immunoblot analysis with anti-GAPDH as a cytoplasmic marker, and anti-RNA polII as a nuclear marker was performed (Fig. 3b).

The gel zymography revealed that only pro-MMP-2 was present in the cytoplasmic fraction of control rat muscle tissue. Its level remained unchanged in post-exercise period. Neither precursor- nor the active form of MMP-9 was detectable in control cytoplasmic extracts, but they became clearly up-regulated upon training (especially the active form) (Fig. 3a). The non active form of MMP-2 was observed in nuclear fraction of normal muscle tissue. Upon training, pro-MMP-2 became increased, and the additional gelatinolytic band appeared, corresponding to an approximate molecular mass of 67 kDa, e.g. the active form of MMP-2. Thus, we conclude that training activated and upregulated MMP-2 in the nuclei. In addition, the nuclear fraction contained the gelatinolytic band of the lower molecular mass that most probably represents an autocatalytically generated active MMP-2 fragment (43 kDa), described by Howard et al. (1991) (see the "Discussion").

To verify the conclusion of gel zymography experiments, we performed regular immunofluorescent stainings for MMP-2 and MMP-9 in the sections of trained muscle. In agreement with the biochemical work we found MMP-2-, but not MMP-9 immunoreactivity in the myonuclei (Fig. 3c–h).

To confront these results with completely different experimental approach, we analyzed the mRNA content of both MMP-2 and MMP-9 in the control and trained muscles. MMPs mRNA levels showed muscle-type specific differences and expression pattern that were generally consistent with the zymography data. As shown in (Fig. 3j), MMP-9 mRNA was only slightly upregulated in Sol muscle after a single bout of exercise, and, in contrast, it was strongly upregulated after 5 days and 2 weeks of repeated physical training. Contrary to the results on MMP-9 mRNA, RT-PCR analyses of MMP-2 mRNA in Sol muscle showed no differences in expression level between control and running groups (Fig. 3j). In EDL, no change in gelatinases mRNAs was detected, except slight, but significant increase in MMP-9 mRNA at 2 h after single bout of exercise.

### Activated, but not quiescent satellite cells are abundant source of MMP-9

Endurance running induces activation of satellite cells in human skeletal muscle (Appell et al. 1988; Kadi and Thornell 1999), and in rodents (Shefer et al. 2010), which is involved in the repair and replacement of injured or necrotic fibers (Carlson and Faulkner 1983) in regard to the post-exercise adaptation and growth (Rosenblatt et al. 1994).

**Fig. 4:**
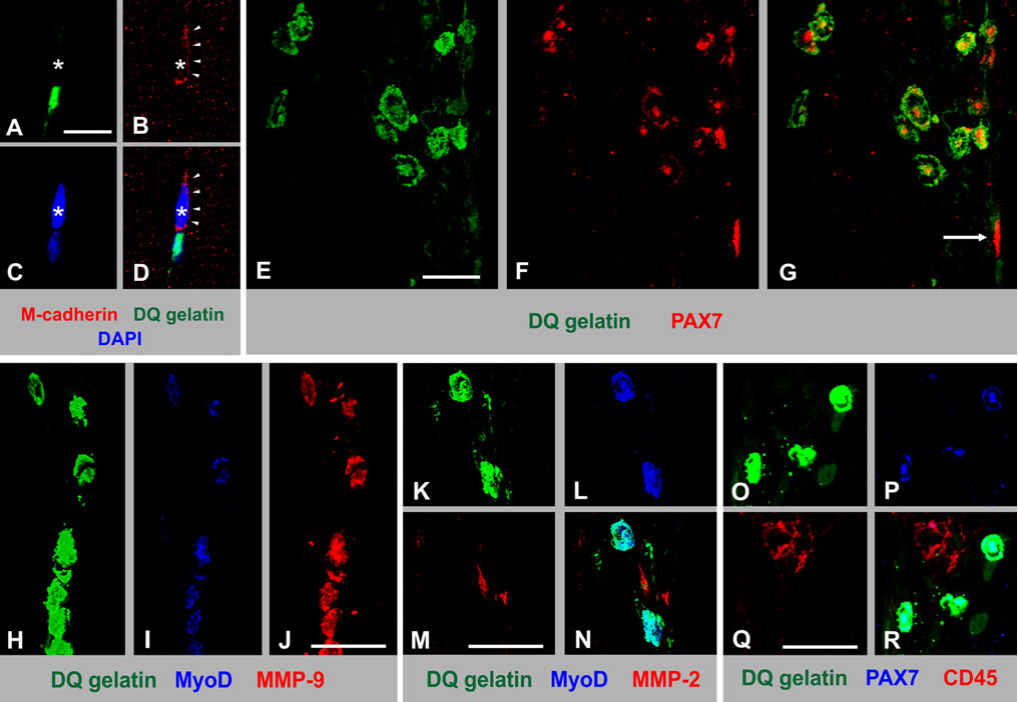
Analysis of gelatinase activity in satellite cells of trained Sol muscle (5 days of running). Confocal maximum projection images demonstrating the absence of gelatinolytic activity (**a**, **d**, *green*) in M-cadherin-immunoreactive (**b**, **d**, *red*, *arrowheads*) quiescent satellite cell (*asterisk*) lying adjacent to a myonucleus containing strong gelatinase activity; nuclei are counterstained with TOPRO dye (**c**, **d**, *blue*). *Scale bar* 20 μm. A group of cells having strong gelatinase activity (**e**, **g**, *green*) and the immunoreactivity of the satellite cell marker PAX7 (**f**, **g**, *red*), lying within the space formerly occupied by the necrotic fiber; the expression of the specific marker, and the positioning of the cells away from the sarcolemma identifies them as activated satellite cells or myoblasts. An *arrow* points to the quiescent PAX7-positive satellite cell adjacent to the muscle fiber; this cell does not contain the gelatinase activity. The images are confocal maximum projections. *Scale bar* 10 μm. A group of strongly gelatinase-postive cells (**h**, *green*) immunoreactive for the specific marker of activated satellite cell/myoblasts MyoD (**i**, *blue*), expressing MMP-9-IR (**j**, *red*). *Scale bar* 25 μm. Confocal maximum projection images demonstrating the absence of MMP-2-IR (**m**, **n**, *red*) in gelatinase-positive (**k**, **n**, *green*), MyoD-immunoreactive (**l**, **n**, *blue*) activated satellite cells/myoblasts. *Scale bar* 25 μm. **o**–**r** Confocal maximum projection images demonstrating that gelatinase-positive (**o**, **r**, *green*), PAX7-immunoreactive (**p**, **r**, *blue*) are distinct from CD45-immunoreactive macrophage-like cells. *Scale bar* 25 μm

To determine whether the nuclei of satellite cells were among those showing increased gelatinase activity after physical training, we co-immunostained the ISZ Sol specimens from 5-day trained animals for satellite cell markers M-cadherin and Pax-7 (Boldrin et al. 2010). The M-cadherin or Pax-7-positive cells lying at the periphery of normal myofibers, representing quiescent satellite cells, did not contain the enzymatic activity, in contrast to myonuclei (Fig. 4a–g, see also inset in Fig. 1h). On the other hand, there were few focal accumulations of cells expressing Pax-7 or MyoD (a marker of activated satellite cells and myoblasts) that had very strong gelatinolytic activity in their cytoplasm (Fig. 4e–g); the cells lied within the apparent remnants of necrotic myofibers, but did not express a pan-leukocyte marker CD45 (Fig. 4o–r). Such cells were found in trained Sol, but not in EDL muscle. We consider these cells to be activated satellite cells or myoblasts that respond to the limited myofiber necrosis, known to occur after several days of intense running. To determine whether these cells express MMP-2 or MMP-9, or both gelatinases, we co-immunostained the ISZ specimens with the antibodies specific for the respective proteins. We found that the very strong gelatinolytic activity of activated satellite cells/myoblasts occurring upon exercise reflects the presence of MMP-9, but not MMP-2 (Fig. 4h–n).

## Discussion

In the present study we investigated the effect of endurance training on the activity and localization of MMP-2 and MMP-9 in Sol and EDL muscles.

To our knowledge, this is the first study on the subcellular localization of MMPs in skeletal muscle upon training. The main findings of our study can be summarized as follows: (1) exercise induces intranuclear activation of MMP-2 in the rat Sol myofibers; (2) there is striking accumulation of active MMP-9 in the cytoplasm of activated satellite cells/myoblasts, associated with foci of necrotic myofibers in exercised Sol.

Recently, in situ zymography technique has been reported to be an accurate, sensitive and quantitative technique for the detection of matrix metalloproteinases activity in various types of cells (Cheng et al. 2008; Gawlak et al. 2009). Therefore, we have undertaken the in situ zymography experiments to study the involvement of the gelatinases in exercise-induced muscle plasticity. Unexpectedly, instead of extracellular deposition, we detected a strong upregulation of gelatinase activity mainly in the nuclei of mature myofibers. This finding was fully confirmed, and extended, by gel zymography combined with subcellular fractionation, showing an upregulation of both the precursor form- and active MMP-2 in nuclear extract of trained rat Sol muscle. On the other hand, by RT-PCR analysis, we detected no increase in MMP-2 mRNA, indicating that the enzyme is just translocated to the nucleus, and is activated therein. We speculate that putative nuclear localization signal carried by pro-MMP-2 on C-terminus should be exposed without the need of pro-peptide cleavage, allowing a transfer of the full-length protein to the nucleus (Cauwe and Opdenakker 2010; Kwan et al. 2004). On the other hand, Sbai et al. (2010) found a potential nuclear localization signal also downstream of the pro-peptide sequence, which suggests that even the active enzyme can be translocated to the nucleus.

The lower molecular weight gelatinolytic band (about 43 kDa) found in the nuclear extracts is most likely an autocatalytically generated active MMP-2 fragment described by Howard et al. (1991). The authors demonstrated that this fragment is robustly generated when MMP-2 is not complexed with its endogenous inhibitor TIMP-2. The virtual absence of 43 kDa band in the cytoplasmic fractions raises an interesting possibility that the activity of MMP-2 in the muscle fibers is differentially controlled by its endogenous inhibitor(s), such as TIMP-2 or TIMP-1, between the nucleus and cytoplasm. Notably, in cultured astrocytes Sbai et al. (2010) detected a significant colocalization of MMP-2 with TIMP-1 in the cytoplasmic vesicles, whereas, they did not report any such colocalization in the nucleus, despite the abundant presence of MMP-2 therein. Alternatively, although much less likely, the lower molecular weight gelatinolytic band could represent the active form of MMP-1 (45 kDa) or MMP-13 (41 kDa), which are capable of cleaving gelatin, albeit with the very low activity (Snoek-van Beurden and Von den Hoff 2005).

Kwan et al. (2004) were the first to demonstrate the presence of MMP-2 within the nucleus of rat cardiac myocytes. MMP-2 was immunoprecipitated from nuclear extracts of heart cells with anti-poly (ADP-ribose) polymerase (PARP) antibody and degraded PARP in vitro (Kwan et al. 2004). PARP is a DNA repair enzyme, which is activated by DNA strand breaks, caused by oxidative stress (Ahmad et al. 2009). The cleavage by MMP-2 would result in PARP inactivation, being protective/beneficial when PARP is overactivated (excessive PARP activity may result in ATP depletion from the cell) or detrimental, e.g. when PARP is needed for DNA repair (Mannello and Medda 2012). Contribution of MMP-2 and MMP-9 to oxidative DNA damage has also been shown by Yang et al. (2010), who detected MMP-2 and MMP-9 in the ischemic neuronal nuclei, and proposed their role in oxidative DNA damage. MMP-3, MMP-13, and MMP-14 have also been reported to accumulate within the nuclei upon noxious stimuli (Cauwe and Opdenakker 2010). Although, we cannot exclude that the phenomenon we observed is related to the limited myofiber damage occurring after repeated strenuous exercise (Kocturk et al. 2008), we do not favor this possibility. The increase in gelatinolytic activity is a very widespread phenomenon, affecting the majority of myofiber nuclei, whereas the necrosis occurs within few small foci. In addition, despite considerable effort, we were unable to detect any sign of DNA damage in the myonuclei using TUNEL technique (not shown).

In the present study, we determined that within the myonuclei, MMP-2 gelatinolytic activity is distributed throughout the nuclear interchromatin area, e.g. outside of the compact chromatin, in the region enriched in active genes and mRNA processing machinery (Markaki et al. 2010). The nuclear upregulation of active MMP-2 after exercise in a strict colocalization with an activated RNA Pol II phosphoepitope suggests a novel role of MMP-2 in nuclear function. MMPs target many intracellular proteins involved in essential housekeeping functions, regulation of transcription and translation, and carbohydrate metabolism (Cauwe and Opdenakker 2010). We propose that nuclear MMP-2 may be implicated in the processes of gene expression in response to training. The similarity of our observations to those by Sbai et al. (2010), performed in astrocytes, showing the localization of MMP-2 immunoreactivity in the interchromatin area, and its nuclear upregulation following cell activation by lipopolysacharide, suggests that the direct involvement of MMPs in activity-dependent gene expression might be a more general phenomenon. The work by Eguchi et al. (2008) showing MMP-3 involvement in transcription of a growth-factor gene in chondrocytes also stands in line with this hypothesis. Interestingly, among several nuclear MMP-3-binding proteins the authors found RNA Pol II (Eguchi et al. 2008). As far as the physiological consequences of MMPs activation inside the cell nucleus are concerned, it is tempting to speculate about the involvement of these enzymes in nuclear architectural remodeling. This higher-order epigenetic phenomenon involves, among other processes, the molecular motors-driven movements of chromatin segments or individual genes, and their gathering in the common transcription factories (Chakalova and Fraser 2010). Such movements should certainly require the release of mechanical constraints imposed by elements of the nucleoskeleton, a quite poorly understood assembly of proteins, formerly referred to as the “nuclear matrix” (Simon and Wilson 2011). Thus, in the nucleus MMPs might work in a way analogous to their major function outside the cell, e.g. the facilitation of cell movements through the extracellular matrix. The verification of some aspects of the aforementioned hypothesis is currently a subject of intense research in our laboratory.

Although our findings are in agreement with the general picture emerging from previous studies on MMP involvement in muscle adaptation to training (Carmeli et al. 2004; Koskinen et al. 2004; Rullman et al. 2009), there are substantial differences between our results and the results of others. For example, Carmeli et al. (2005) found no changes in MMP-9 expression level in rat hindlimb muscles in response to 14-day long high-intensity treadmill running; the authors noticed the increase in MMP-2 expression, but in contrast to our observations, this occurred only in muscles with high percentage of fast glycolytic fibers (gastrocnemius, superficial quadriceps), and not in Sol. The discrepancies between the results of aforementioned study and our data may be related to the differences in exercise protocols, such as longer habituation period (3 days prior beginning) and gradually increased loading (20 min of running at the beginning with daily increases of 10 min until 50 min during 2 weeks) used by Carmeli and colleagues, or to differences in strain, sex and age of the animals. According to another study, an increased level and/or activity of MMP-2, but not MMP-9, was observed in rat Sol and quadriceps femoris muscles at 2, 4, 7 days after acute downhill exercise (Koskinen et al. 2001), which is partially consistent with our findings. It is even more difficult to compare the results of human studies to the ones obtained in animal models, however, in agreement with us, Rullman et al. (2007, 2009) found an increase in expression/activity of both MMP-2 and MMP-9 after endurance exercise in vastus lateralis muscle. Similar to us, these authors found evidence that MMP-2 is expressed by myofibers (Rullman et al. 2009). The concept of MMP-involvement in adaptation to training appears to gain support by the recent study of Dahiya et al. (2011), who generated transgenic mice expressing constitutively active MMP-9 in skeletal muscle. The mutant animals had increased proportion of fast-twitch fibers in Sol muscle, increased levels of contractile proteins and general hypertrophy of the skeletal muscle (Dahiya et al. 2011).

In the present study, we found that the increase in MMP expression/activity upon exercise occurs almost exclusively in Sol, and not EDL. This may result from their physiological, and/or structural properties, and muscle recruitment pattern during running. Motor units of Sol are highly recruited during locomotory activities (Smith et al. 2001) and have, more frequently than EDL muscle, micro-lesions (Irintchev and Wernig 1987; Smith et al. 1997, 2001). With regards to signaling by mitogen-activated protein kinases (MAPK), which are potent inducers of MMP activity and/or expression (Chakraborti et al. 2003), Sol has been found to express higher levels of ERK1/2 and p38 MAPK, than EDL (Wretman et al. 2000). An increased exercise-induced activation of both kinases has been found in Sol after endurance training (Lee et al. 2002).

An intense treadmill running is known to cause focal myofiber necrosis in Sol, but not EDL muscles (Irintchev and Wernig 1987). Such myofiber damage leads to an activation, proliferation and differentiation of muscle satellite cells, resulting in the appearance of small regenerating fibers (Irintchev and Wernig 1987; Smith et al. 1997). Here, we have found that activated (MyoD-positive) satellite cells, or myoblasts, occupying the space of necrotic myofibers in Sol had very strong gelatinolytic activity, due to the high expression of MMP-9. These cells could be the major source of cytoplasmic MMP-9 detected by our gel zymography in cytoplasmic fractions of exercised Sol, although we cannot totally rule out the possibilty that some MMP-9 is produced also in mature myofibers. However, if present, such an amount is below the sensitivity of our immunocytochemical detection that showed no MMP-9-IR in mature myofibers above the background level (not shown). The finding of high MMP-9 expression in activated satellite cells/myoblasts is in complete agreement with the studies showing MMP-9 playing a role in the early phase of muscle repair, and with in vitro experiments demonstrating the involvement of this enzyme in myoblast differentiation (reviewed by Chen and Li 2009; see also Zimowska et al. 2008). Interestingly, MMP-2 is considered to be involved in the later stages of differentiation, such as myoblast fusion (Zimowska et al. 2008).

In conclusion, our study identifies, for the first time, the cellular and subcellular compartments of the skeletal muscle in which the gelatinase activities are regulated by training. In particular, we demonstrate the training-induced activation of MMP-2 in myonuclei, suggesting a novel role of this enzyme in exercise-evoked nuclear events, such as gene expression. Our results are in line with the results of numerous other studies pointing to important role of MMPs in skeletal muscle physiology and pathophysiology, and to potential clinical applications of their therapeutic manipulation (reviewed by Carmeli et al. 2004; see also Kumar and Bhatnagar 2010 and references therein).

## Electronic supplementary material

Below is the link to the electronic supplementary material.

Suppl. Fig. *The representation of a semi*-*automatic algorithm used for quantitative analysis of the myonuclear gelatinolytic activity in sections subjected to* in situ *zymography.* The analysis is performed in the images (A as an example) of the specimens triple-labeled for (i) β-dystroglycan, a marker of sarcolemma (Alexa 555, red in A), (ii) gelatinolytic activity (fluorescein-conjugated DQ-gelatin, green in A), and (iii) nuclei (DAPI, blue in A), acquired through the confocal microscope. At the first step the three-channel 24-bit RGB image (A), is being split into the three individual 8-bit images (B-D), corresponding to the different channels. To measure the myonuclear activity specifically, the red and blue channels (B and C respectively) are being merged (E), to identify and manually erase all nuclei situated outside the sarcolemma, using the appropriate black drawing tool (F). Then the edited red-blue image (F) is being split again, and the channel containing only the myonuclei (G) is binarized (H) after applying the default automatic threshold if ImageJ. The binary image (H) is then inverted (J), and subtracted from the DQ-gelatin-containing channel (D). As a result, the image representing only the myonuclear activity (everything else black) is generated, subsequently subjected to the “analyze particles” function of the ImageJ, that produces the mean myonuclear activity. Only the particles with the area larger than 5 pixels are taken into account, to exclude the noise. (TIFF 2784 kb)

## Abbreviations

DAPI: 4,6-Diamidino-2-phenylindole
DQ: Dye-quenched
ECM: Extracellular matrix
EDL: Extensor digitorium longus
GAPDH: Glyceraldehyde 3-phosphate dehydrogenase
IR: Immunoreactivity
ISZ: In situ zymography
MAPK: Mitogen activated protein kinase
MMP: Matrix metalloproteinase
MyoD: Myogenic differentiation-1
PARP: Poly (ADP-ribose) polymerase
Pax-7: Paired box protein
Sol: Soleus
Tissue inhibitor of metalloperoteinases: TIMP
TUNEL: Terminal deoxynucleotidyl transferase mediated dUTP nick end labeling assay
RNA polII: RNA polymerase II
