# Supplementary figures and images for: Fine-structural distribution of MMP-2 and MMP-9 activities in the rat skeletal muscle upon training: a study by high-resolution in situ zymography
Source: Histochem Cell Biol. 2012 Mar 15;138(1):75–87. doi: 10.1007/s00418-012-0940-5 (PMC3374103; doi:10.1007/s00418-012-0940-5)

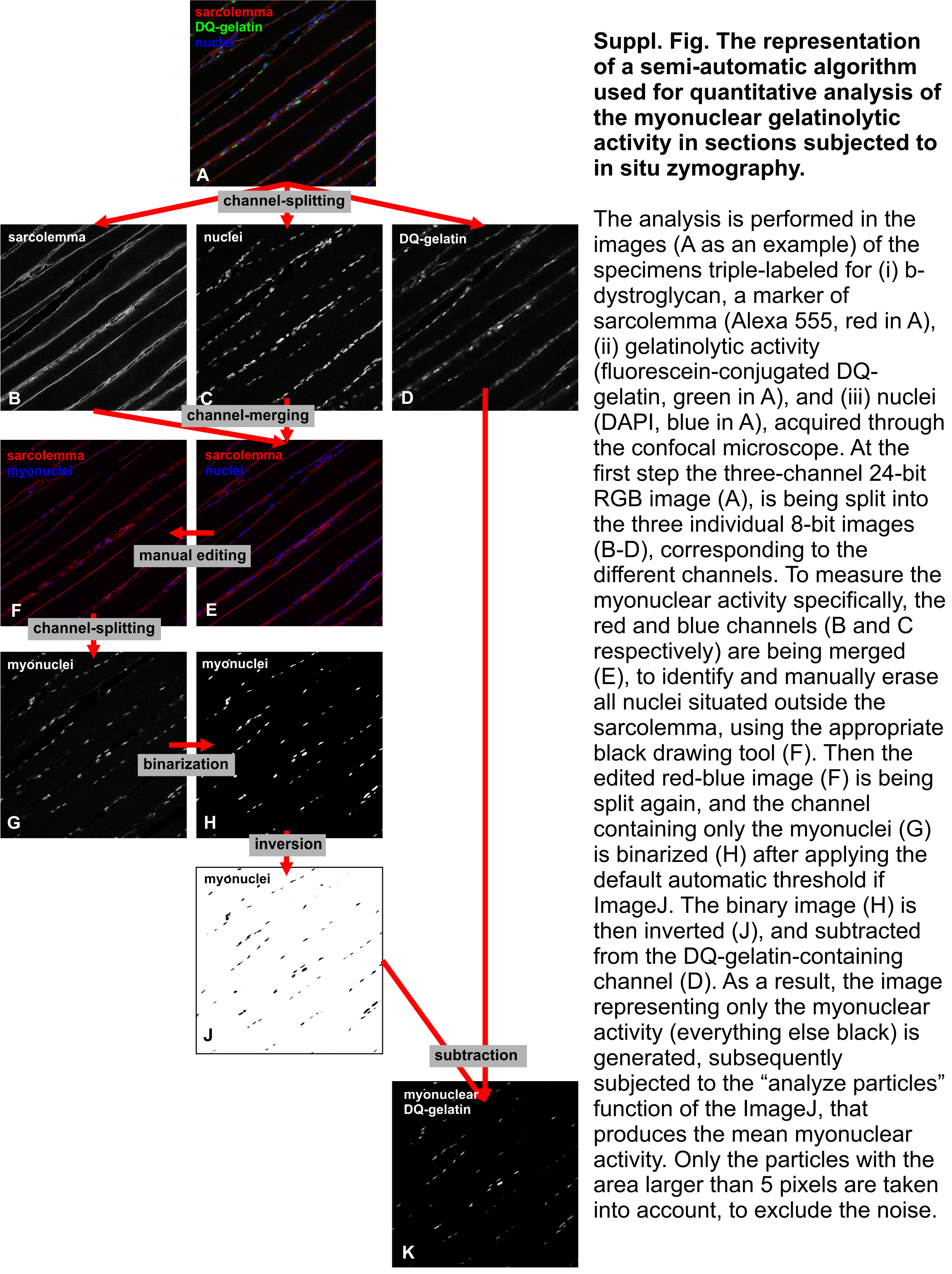

Supplement: Supplementary file 1 — Suppl. Fig. The representation of a semi-automatic algorithm used for quantitative analysis of the myonuclear gelatinolytic activity in sections subjected to in situ zymography. The analysis is performed in the images (A as an example) of the specimens triple-labeled for (i) β-dystroglycan, a marker of sarcolemma (Alexa 555, red in A), (ii) gelatinolytic activity (fluorescein-conjugated DQ-gelatin, green in A), and (iii) nuclei (DAPI, blue in A), acquired through the confocal microscope. At the first step the three-channel 24-bit RGB image (A), is being split into the three individual 8-bit images (B-D), corresponding to the different channels. To measure the myonuclear activity specifically, the red and blue channels (B and C respectively) are being merged (E), to identify and manually erase all nuclei situated outside the sarcolemma, using the appropriate black drawing tool (F). Then the edited red-blue image (F) is being split again, and the channel containing only the myonuclei (G) is binarized (H) after applying the default automatic threshold if ImageJ. The binary image (H) is then inverted (J), and subtracted from the DQ-gelatin-containing channel (D). As a result, the image representing only the myonuclear activity (everything else black) is generated, subsequently subjected to the “analyze particles” function of the ImageJ, that produces the mean myonuclear activity. Only the particles with the area larger than 5 pixels are taken into account, to exclude the noise. (TIFF 2784 kb) [file 418_2012_940_MOESM1_ESM.tif]
